# A nomogram model to predict cognitive impairment in patients with spontaneous intracerebral hemorrhage

**DOI:** 10.3389/fneur.2026.1763371

**Published:** 2026-03-03

**Authors:** Yin Ren, Peimin Yu, Suihan Ye, QingYi Han, Xianglong Song, Liechi Yang

**Affiliations:** The Affiliated Hospital of Xuzhou Medical University, Xuzhou, China

**Keywords:** cognitive impairment, nomogram, pulmonary infection, spontaneous intracerebral hemorrhage, years of education

## Abstract

**Purpose:**

Post-stroke cognitive impairment (PSCI) after spontaneous intracerebral hemorrhage (sICH) is highly prevalent and severely impacts patients’ long-term quality of life. However, accurate prediction tools that integrate acute-phase complications with sociodemographic characteristics are currently lacking. This study aimed to identify independent risk factors for PSCI in sICH patients and to construct a visual nomogram prediction model to guide clinical risk stratification prior to hospital discharge.

**Methods:**

We retrospectively analyzed clinical data from 264 sICH patients admitted to the Affiliated Hospital of Xuzhou Medical University between July 2023 and July 2025. Patients were classified into cognitive impairment and cognitively normal groups based on the Montreal Cognitive Assessment (MoCA) score (<22). The dataset was randomly split into a training set (*n* = 198, 75%) and a validation set (*n* = 66, 25%). Univariate and multivariate logistic regression analyses were employed to screen for independent predictors, which were then used to construct the nomogram model. The model’s discriminative ability, calibration, and clinical utility were validated using the area under the receiver operating characteristic curve (AUC), calibration curves, and decision curve analysis (DCA).

**Results:**

The overall incidence of PSCI in this cohort was 44.3%. Multivariate logistic regression analysis identified pulmonary infection (OR 3.980, 95% CI 2.075–7.635, *p* = 0.002) and hematoma volume (OR 1.030, 95% CI 1.015–1.045, *p* < 0.001) as independent risk factors for PSCI, whereas years of education (OR 0.885, 95% CI 0.831–0.944, *p* < 0.001) served as an independent factor associated with reduced risk. The nomogram model demonstrated excellent discriminative ability with AUCs of 0.771 and 0.820 in the training and validation sets, respectively. Calibration curves indicated high consistency between predicted probabilities and observed outcomes. DCA confirmed clinical net benefit across a wide range of threshold probabilities.

**Conclusion:**

This study successfully developed a nomogram prediction model incorporating pulmonary infection, hematoma volume, and years of education. The model suggests that cognitive decline after sICH is associated with a combination of systemic inflammation (brain–body axis interaction), primary structural injury, and insufficient cognitive reserve. This user-friendly and accurate scoring tool can assist clinicians in identification of high-risk subgroups for PSCI upon completion of inpatient care, thereby informing intensified clinical monitoring and rehabilitation planning.

## Introduction

Spontaneous intracerebral hemorrhage (sICH) is a catastrophic cerebrovascular event with high global mortality and disability rates, accounting for approximately 28% of all stroke types and imposing a particularly heavy burden in low- and middle-income countries (1, 2). Although acute survival rates have improved significantly with advances in neurocritical care and minimally invasive surgical techniques, survivors often face long-term neurological deficits, encompassing not only motor and sensory impairments but also severe cognitive decline (3). Among these, post-stroke cognitive impairment (PSCI), an often-overlooked “invisible disability,” is highly prevalent among sICH survivors. Recent large cohort studies and systematic reviews report acute-phase incidence rates as high as 84%, with a dynamic evolution over time that significantly increases the risk of conversion to dementia (4). The loss of cognitive function not only deprives patients of independent living but also substantially increases the caregiving burden on families and society, posing a major public health challenge.

Current academic opinion increasingly favors the view that cognitive decline after sICH is a complex, multifactorial pathological process rather than a consequence of simple neuroanatomical injury. Previous studies have identified several risk factors associated with poor cognitive outcomes, primarily focusing on non-modifiable indicators such as advanced age, prior stroke history, and specific neuroimaging features (e.g., hematoma volume, white matter hyperintensity burden, cerebral microbleeds, and cortical superficial siderosis) (5–7). Furthermore, genetic background, such as APOE genotype, has also been linked to long-term cognitive outcomes. In recent years, growing evidence suggests that systemic factors may influence central nervous system recovery through complex “brain–body axis” interactions. In particular, systemic inflammatory responses triggered by infection are considered a crucial bridge connecting acute brain injury with long-term neurodegeneration (8). Concurrently, based on the cognitive reserve (CR) theory, education level and socioeconomic status significantly modulate cognitive resilience following brain injury, which is especially pertinent in aging societies (9).

Although several predictive models for cognitive impairment have been developed for ischemic stroke or traumatic brain injury (TBI) (10), studies specifically targeting the sICH patient population are relatively scarce. Existing scoring systems for sICH often focus on predicting mortality or limb motor function, frequently failing to adequately encompass the complex variables contributing to cognitive decline (11). Notably, existing prediction models often neglect the comprehensive consideration of acute-phase complications and sociodemographic characteristics, factors that are frequently modifiable or require clinical focus (12). Additionally, some prior studies have limited sample sizes or did not employ strict criteria to exclude the confounding effect of pre-existing cognitive impairment, limiting the generalizability of their conclusions for guiding precise clinical decisions. Therefore, this study aims to construct and validate a nomogram risk prediction model designed as an “in-hospital update model.” By integrating admission baseline indicators with complications emerging during hospitalization (specifically pulmonary infection), this model translates complex clinical variables into a simple, visual assessment tool. It enables clinicians to perform individualized stratification of long-term cognitive prognosis at the time of discharge, thereby informing the development of precise neuroprotective strategies and early cognitive rehabilitation plans.

## Materials and methods

### Study design

We conducted a retrospective clinical study using data from the Affiliated Hospital of Xuzhou Medical University. The study period spanned from July 2023 to July 2025. The primary aim was to investigate risk factors for cognitive impairment in patients with spontaneous intracerebral hemorrhage (sICH) and to construct a corresponding predictive nomogram model. The prediction time point was defined as the time of hospital discharge. Consequently, the model variables include both baseline characteristics available at admission and clinical complications (e.g., pulmonary infection) that developed during the hospital stay. The study protocol adhered to the ethical principles of the Declaration of Helsinki, with ethical approval number: XYFY2025-KL670-01.

### Patient selection

**Figure 1:**
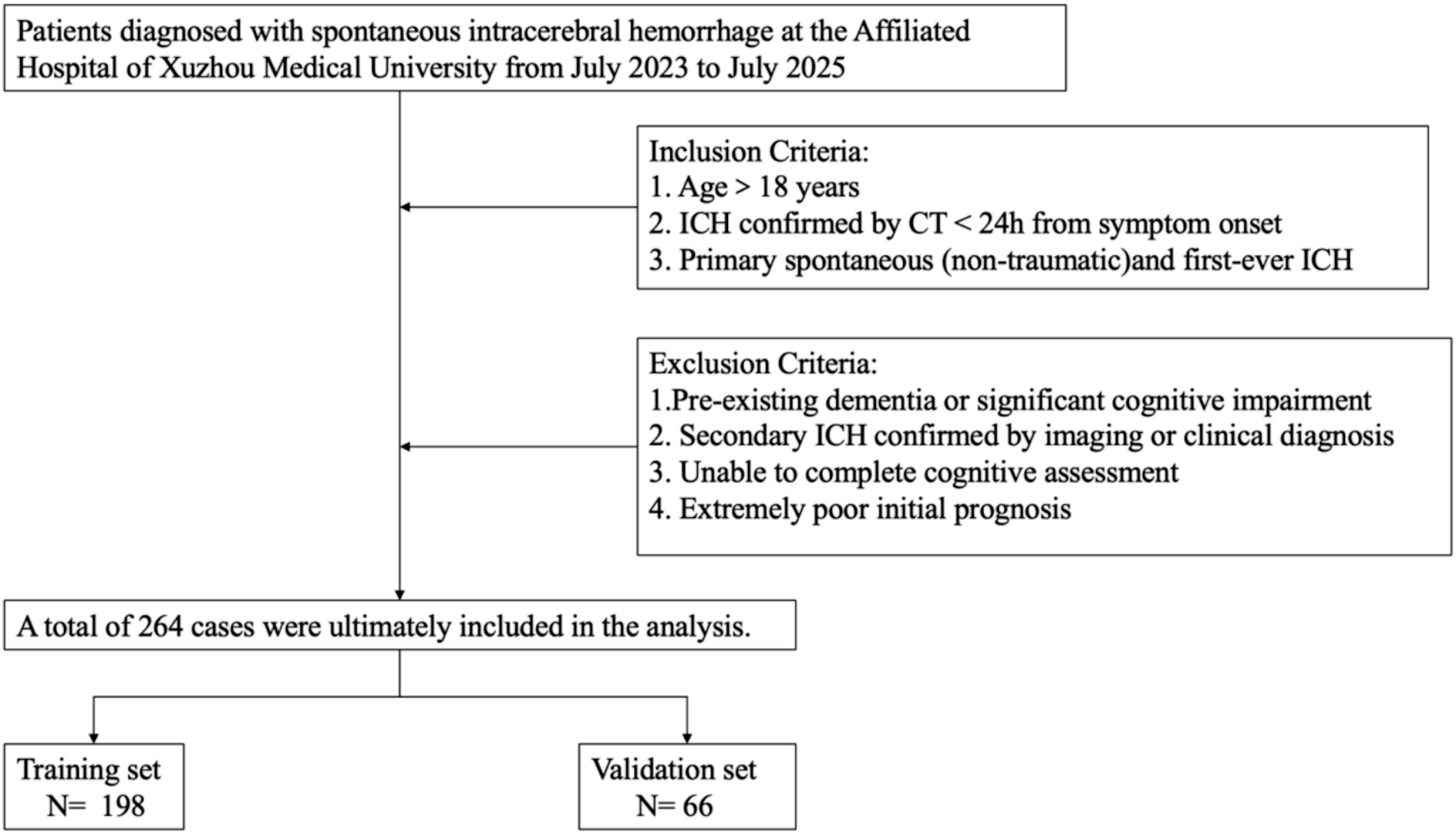
The flowchart of patient selection.

All patients were diagnosed with intracerebral hemorrhage via CT scan within 24 h of symptom onset. sICH was defined according to the AHA/ASA 2022 guidelines (11). To ensure cohort homogeneity, we strictly included patients aged ≥18 years with a first-ever episode of primary spontaneous (non-traumatic) ICH. To minimize confounding factors, the following exclusion criteria were applied: (1) history of dementia or significant pre-existing cognitive deficits; (2) secondary ICH confirmed clinically or radiologically (e.g., vascular malformation, tumor); (3) inability to complete cognitive assessment due to severe clinical condition; (4) extremely poor initial prognosis severely affecting survival. Ultimately, 264 eligible patients were identified (Figure 1). The cohort was randomly divided into a training set (*n* = 198, 75%) for model development and a validation set (*n* = 66, 25%) for internal validation using a random number generator.

### Data extraction and outcome definition

Data extraction was performed via the hospital’s electronic medical record system. We compiled a comprehensive dataset covering demographic information (age, sex, years of education), admission vital signs [body temperature, mean arterial pressure (MAP)], and baseline health indicators [e.g., body mass index (BMI) and length of hospital stay]. Clinical severity was assessed using the Glasgow Coma Scale (GCS) score and calculated hematoma volume. Baseline laboratory parameters were derived from the first peripheral venous blood samples collected immediately upon hospital admission (strictly within 24 h of symptom onset) to reflect the physiological state prior to in-hospital medical intervention. These parameters were obtained from the institution’s standardized emergency admission panels. Specifically, mean corpuscular hemoglobin concentration (MCHC) was extracted as a standard calculated component of the routine complete blood count (CBC). Gamma-glutamyl transferase (γ-GT) was extracted as part of the routine hepatic function profile, which is standardly ordered at admission to screen for comorbidities such as alcohol use disorder. The comprehensive panel also included hemoglobin, C-reactive protein (CRP), creatinine (Cr), albumin, potassium (K^+^), D-dimer, and random blood glucose (GLU). Inflammatory indices, namely the neutrophil-to-lymphocyte ratio (NLR) and platelet-to-lymphocyte ratio (PLR), were calculated. Treatment-related variables included optimal intervention timing, surgical method (conservative management, craniotomy, or trepanation and drainage), and specific hemorrhage location (basal ganglia, cerebral cortex, or thalamus). Additionally, comorbidities (hypertension, diabetes, epilepsy, prior cerebral infarction) and complications (pulmonary infection, intracranial infection, need for tracheotomy) were recorded. The primary endpoint was the occurrence of cognitive impairment. This study is a retrospective analysis of data generated through the routine Stroke center quality control pathway of our institution. This clinical standard of care mandates that all survivors of cerebrovascular events (including sICH) undergo a comprehensive face-to-face assessment at 3 months post-discharge. During this visit, trained neurologists routinely administer the Montreal Cognitive Assessment (MoCA). We retrospectively extracted these pre-existing clinical data for the current analysis. Cognitive impairment was defined as a MoCA score <22.

### Statistical analysis and model construction

Candidate predictor variables were pre-specified based on their established clinical relevance to post-stroke cognitive outcomes in the literature and their availability at the time of hospital discharge, aligning with the intended use of the model. These included demographic factors (age, sex, years of education), indices of initial injury severity (hematoma volume, Glasgow Coma Scale score), key laboratory markers (C-reactive protein, neutrophil-to-lymphocyte ratio), and clinically significant in-hospital complications (pulmonary infection). For baseline characteristics, normally distributed continuous variables are presented as mean ± standard deviation (SD) and compared using Student’s *t*-test; non-normally distributed variables are presented as median and interquartile range (IQR) and compared using the Mann–Whitney *U* test. Categorical data are summarized as frequencies and percentages, compared using the chi-square test or Fisher’s exact test. To mitigate the risk of overfitting associated with traditional univariate screening followed by multivariate regression, we employed the least absolute shrinkage and selection operator (LASSO) regression for variable selection within the training set. The optimal tuning parameter (lambda) was determined via 10-fold cross-validation based on the minimum criterion. Variables with non-zero coefficients retained by LASSO were then entered into a standard multivariate logistic regression model to obtain the final odds ratios and construct the nomogram. This approach balances model parsimony with predictive stability. Model performance was rigorously evaluated across three dimensions: discrimination, calibration, and clinical utility. Discrimination was quantified using the area under the receiver operating characteristic (ROC) curve (AUC). Calibration curves were plotted to visualize the agreement between predicted probabilities and observed outcomes. Finally, decision curve analysis (DCA) was employed to determine the clinical net benefit of using the nomogram across different threshold probabilities. To obtain a more robust and stable estimate of the final model’s performance and to mitigate the variability inherent in a single split, we further performed repeated 10-fold cross-validation exclusively on the training set using the fixed predictors identified by LASSO.

### Data completeness

All variables included in the analysis were extracted from mandatory fields in the hospital’s structured electronic medical record system and the standardized 3-month follow-up protocol. Consequently, there were no missing data for any of the demographic, clinical, laboratory, or outcome variables analyzed in this cohort of 264 patients. A complete-case analysis was therefore inherently performed.

Statistical analyses were performed using R software (version 4.2) and SPSS (version 25.0). A two-sided *p*-value <0.05 was defined as statistically significant.

## Results

### Study cohort characteristics

The final analysis included 264 patients diagnosed with spontaneous intracerebral hemorrhage (Figure 1). Within this cohort, 117 patients (44.3%) were identified as having cognitive impairment. Comparative analysis between the training set (*n* = 198) and validation set (*n* = 66) showed balanced distribution of baseline characteristics (Table 1). No statistically significant differences were observed between the two sets regarding demographics (e.g., age, sex, years of education) or clinical indicators (e.g., hematoma volume, GCS score) (*p* > 0.05), confirming the validity of the split. The prevalence of cognitive impairment was 47.0% in the training group and 36.4% in the validation group, with no significant difference (*p* = 0.174).

**Table 1:** Baseline characteristics of patients with spontaneous intracerebral hemorrhage.

Variable	Total (*n* = 264)	Training (*n* = 198)	Validation (*n* = 66)	*p*-value
Cognitive impairment, *n* (%)				0.174
No	147 (55.7)	105 (53)	42 (63.6)	
Yes	117 (44.3)	93 (47)	24 (36.4)	
Age	61 (54, 69)	61 (53, 69)	61 (56, 68)	0.343
Hospital stays	16 (11, 24)	16 (12, 23)	15 (11, 25)	0.513
Years of education	9 (3, 9)	9 (3, 9)	9 (3, 9)	0.895
Hematoma volume	21.0 (6.9, 40.0)	21.1 (7.5, 40.0)	20.7 (5.7, 41.3)	0.628
GCS score	12 (6, 15)	12 (6, 14)	13 (7, 15)	0.107
BMI	24.99 (23.1, 27.5)	25.0 (23.1, 27.3)	25.4 (22.9, 27.9)	0.689
Temperature	36.7 (36.5, 37.0)	36.7 (36.5, 37.0)	36.6 (36.5, 37.0)	0.968
MAP	115.0 (104.7, 126.6)	115.0 (103.5, 125.5)	118.2 (108.2, 130.4)	0.104
Optimal timing for intervention therapy	12.0 (7, 25)	12.2 (7, 26)	10 (7, 25)	0.547
GLU	7.0 (6.1, 8.5)	6.9 (6.2, 8.5)	7.3 (6.1, 8.4)	0.945
NLR	5.8 (3.6, 12.5)	5.7 (3.4, 12.0)	6.9 (3.3, 13.7)	0.437
Hemoglobin	137 (125, 148)	136 (124, 146)	141 (128, 151)	0.083
MCHC	328 (322, 336)	328 (321, 336)	331 (323, 338)	0.124
PLR	187.1 (130.2, 280.4)	188.8 (130.0, 272.7)	181.8 (132.2, 304.6)	0.924
CRP	1.7 (0.6, 6.9)	1.6 (0.6, 6.1)	2.1 (0.7, 9.1)	0.231
Cr	56.0 (47.0, 69.8)	55 (47, 69)	58 (47, 72)	0.629
γ-GT	20.0 (14.3, 32.0)	21 (15, 32)	20 (14, 37)	0.655
Albumin	42.0 (39.0, 44.3)	42.0 (39.0, 44.3)	41.6 (38.6, 43.9)	0.463
K^+^	3.8 (3.5, 4.1)	3.8 (3.6, 4.1)	3.8 (3.5, 4.1)	0.999
D-dimer	0.5 (0.2, 1.1)	0.5 (0.3, 1.1)	0.4 (0.2, 1.5)	0.552
Gender				0.100
Female	92 (34.8)	75 (37.9%)	17 (25.8%)	
Male	172 (65.2)	123 (62.1%)	49 (74.2%)	
Surgical method				0.718
Conservative treatment	132 (50.0)	99 (50.0)	33 (50.0)	
Craniotomy	76 (28.8)	59 (29.8)	17 (25.8)	
Trepanation and drainage	56 (21.2)	40 (20.2)	16 (24.2)	
Bleeding location				0.942
Basal ganglia	80 (30.3)	61 (30.8)	19 (28.8)	
Cerebral cortex	123 (46.6)	92 (46.5)	31 (47)	
Thalamus	61 (23.1)	45 (22.7)	16 (24.2)	
Hypertension				0.527
No	51 (19.3)	36 (18.2%)	15 (22.7%)	
Yes	213 (80.7)	162 (81.8%)	51 (77.3%)	
Diabetes				0.923
No	221 (83.7)	165 (83.3%)	56 (84.8%)	
Yes	43 (16.3)	33 (16.7%)	10 (15.2%)	
Cerebral infarction				0.108
No	181 (68.6)	130 (65.7%)	51 (77.3%)	
Yes	83 (31.4)	68 (34.3%)	15 (22.7%)	
Epilepsy				0.200
No	250 (94.7)	185 (93.4%)	65 (98.5%)	
Yes	14 (5.3)	13 (6.6%)	1 (1.5%)	
Pulmonary infection				0.131
No	191 (72.3)	138 (69.7%)	53 (80.3%)	
Yes	73 (27.7)	60 (30.3%)	13 (19.7%)	
Intracranial infection				0.416
No	256 (96.6)	193 (97.5%)	63 (95.5%)	
Yes	8 (3.4)	5 (2.5%)	3 (4.5%)	
Tracheotomy				0.918
No	227 (86.0)	171 (86.4%)	56 (84.8%)	
Yes	37 (14.0)	27 (13.6%)	10 (15.2%)	
Smoking				1
No		133 (67.2%)	45 (68.2%)	
Yes		65 (32.8%)	21 (31.8%)	

BMI, body mass index; GCS, Glasgow Coma Scale; MAP, mean arterial pressure; WBC, white blood cell count; Glu, glucose; Cr, creatinine; γ-GT, gamma-glutamyl transferase; CRP, C-reactive protein; NLR, neutrophil-to-lymphocyte ratio; PLR, platelet-to-lymphocyte ratio; MCHC, mean corpuscular hemoglobin concentration.

### Screening of independent risk factors

**Figure 2:**
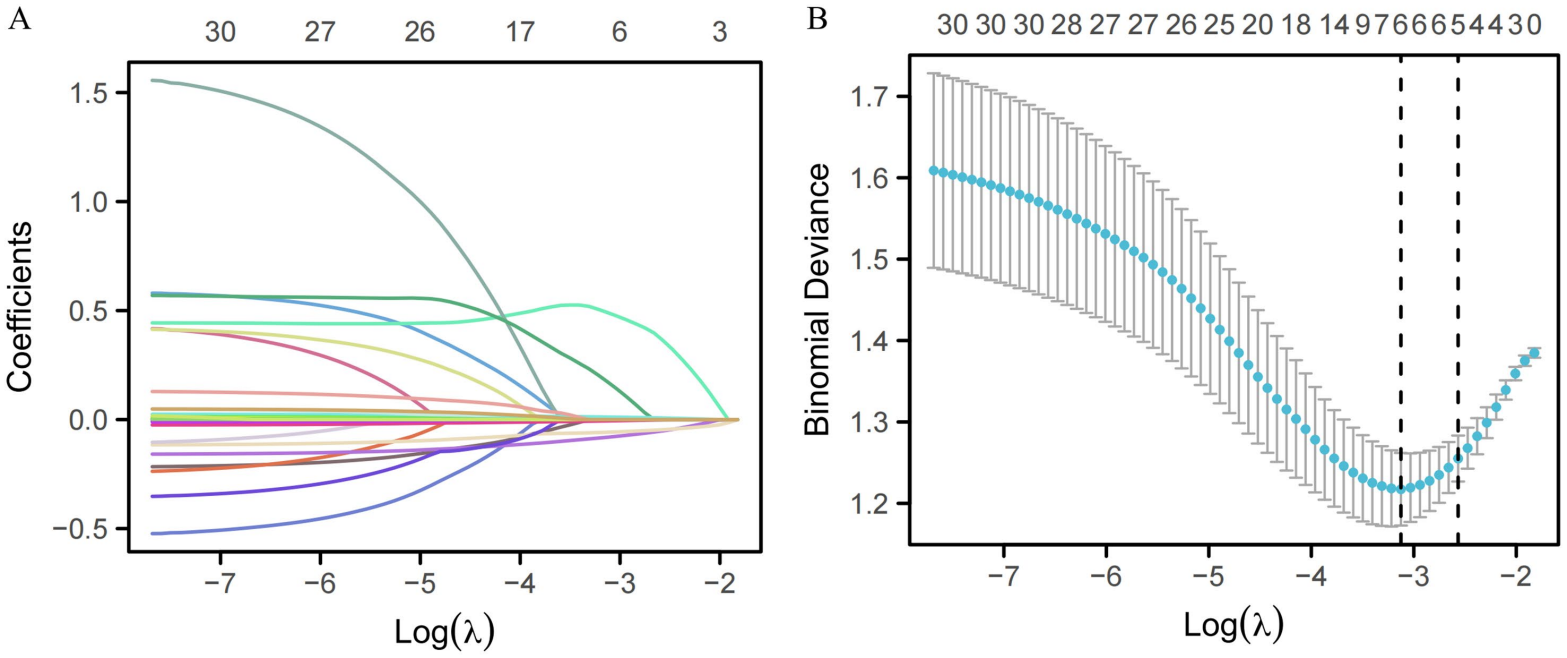
Variable selection using the LASSO binary logistic regression model. **(A)** LASSO coefficient profiles of the candidate clinical features. A coefficient profile plot was generated against the log (lambda) sequence. **(B)** Tuning parameter (lambda) selection in the LASSO model using 10-fold cross-validation. The binomial deviance curve is plotted against log (lambda). Vertical dotted lines indicate the optimal lambda values based on the minimum criteria and the 1-standard error (1-SE) criteria, resulting in the identification of five features with non-zero coefficients.

Initial screening via univariate logistic regression in the training set identified several factors significantly associated with cognitive decline (*p* < 0.05), including hemorrhage location, length of hospital stays, pulmonary infection, tracheotomy, years of education, GCS score, hematoma volume, and hemoglobin level (Table 2). To address potential multicollinearity among these variables and prevent overfitting, we employed LASSO regression for rigorous feature selection. This penalized shrinkage method identified five variables with non-zero coefficients as the most robust predictors: pulmonary infection, years of education, hematoma volume, GCS score, and hemoglobin (Figure 2). These five candidate variables were subsequently entered into a multivariate logistic regression model (Table 3). Ultimately, three variables remained as independent predictors of cognitive impairment: pulmonary infection (OR = 3.028, 95% CI 1.480–6.166, *p* = 0.002), years of education (OR = 0.868, 95% CI 0.809–0.931, *p* < 0.001), and hematoma volume (OR = 1.030, 95% CI 1.014–1.047, *p* < 0.001). The results are consistent with those obtained after conducting a univariate logistic regression analysis and including the significant variables in a multivariate logistic regression. The longer education demonstrated a protective effect, whereas pulmonary infection and larger hematoma volume were associated with an increased risk of cognitive impairment.

**Table 2:** Univariate logistic regression analysis of predictive variables for cognitive impairment in the training cohort.

Variable	OR	95% CI	*p*-value
Gender	0.663	0.372–1.180	0.162
Age	1.020	0.996–1.044	0.108
BMI	0.977	0.910–1.050	0.529
Temperature	1.593	0.862–2.946	0.137
MAP	1.003	0.988–1.019	0.668
Bleeding location	0.690	0.497–0.958	0.026
Hospital stays	1.037	1.008–1.066	0.011
Pulmonary infection	3.980	2.075–7.635	0.000
Tracheotomy	4.812	1.849–12.528	0.001
Years of education	0.885	0.831–0.944	0.000
GCS score	0.845	0.784–0.910	0.000
Hematoma volume	1.030	1.015–1.045	0.000
Hemoglobin	0.973	0.955–0.991	0.003
Optimal timing for intervention therapy	1.000	0.994–1.006	0.995
GLU	1.054	0.970–1.145	0.214
NLR	1.023	0.990–1.057	0.173
MCHC	0.992	0.966–1.019	0.563
PLR	1.002	1.000–1.005	0.052
CRP	1.001	0.989–1.013	0.878
Cr	0.989	0.977–1.002	0.091
γ-GT	1.001	0.997–1.004	0.788
Albumin	1.005	0.990–1.021	0.514
K^+^	0.785	0.413–1.491	0.460
D-dimer	1.042	0.961–1.131	0.321
Treatment	1.381	0.963–1.980	0.079
Hypertension	1.496	0.715–3.127	0.285
Diabetes	1.244	0.589–2.629	0.567
Cerebral infarction	1.203	0.669–2.166	0.537
Epilepsy	0.689	0.217–2.184	0.527
Intracranial infection	4.674	0.513–42.587	0.171
Smoking	0.792	0.463–1.438	0.443

**Table 3:** Multivariate logistic regression analysis of independent predictors of cognitive impairment in the training cohort.

Variables	*β*	SE	Wald	*p*-value	OR (95% CI)
Pulmonary infection	1.108	0.363	9.337	0.002	3.028 (1.480–6.166)
Years of education	−0.142	0.036	15.685	<0.001	0.868 (0.809–0.931)
Hematoma volume	0.030	0.008	12.721	<0.001	1.030 (1.014–1.047)
Constant	−0.306	0.326	0.878	0.349	0.737

### Development and validation of the nomogram

**Figure 3:**
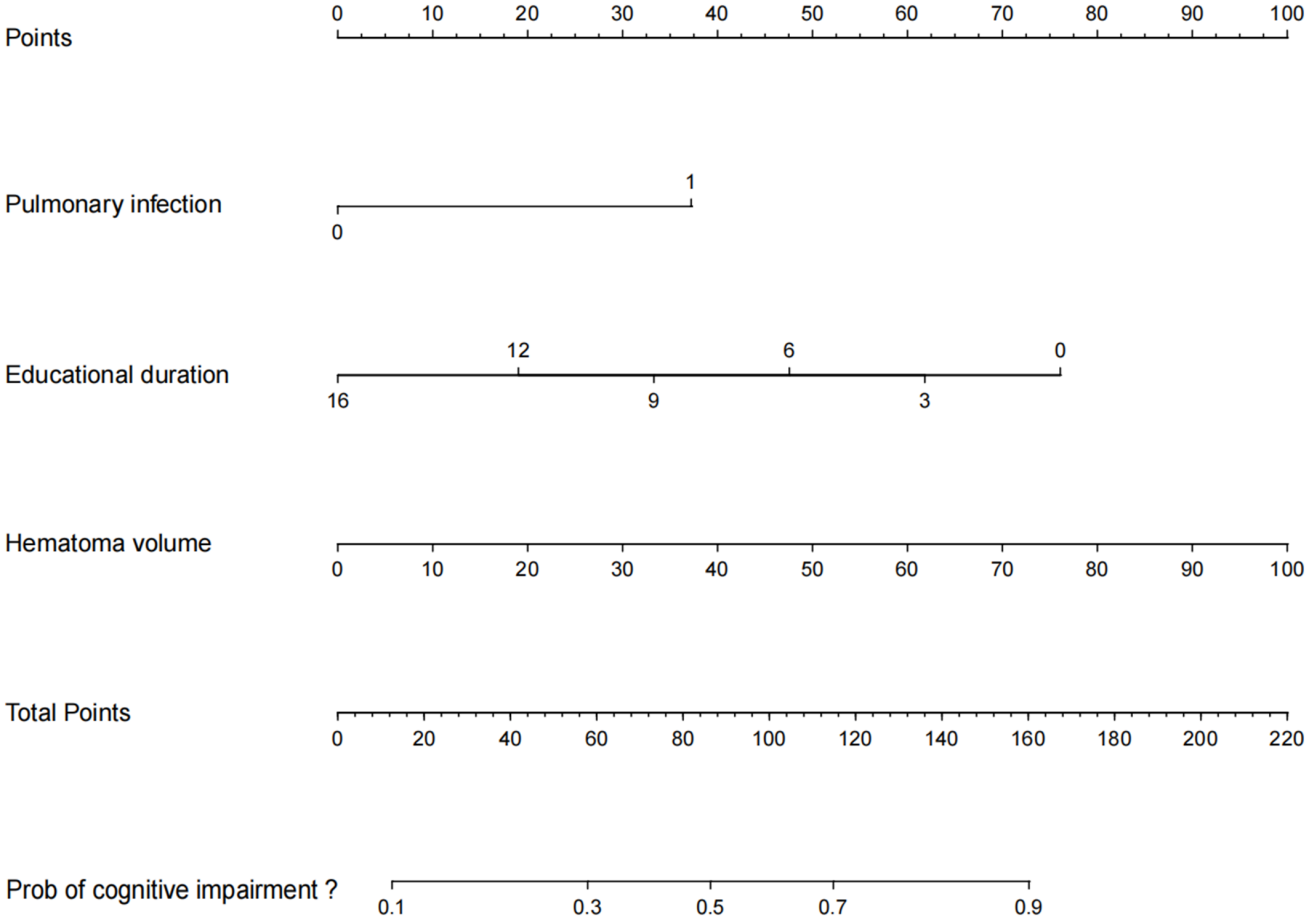
Nomogram to estimate the risk of MoCA in patients.

**Figure 4:**
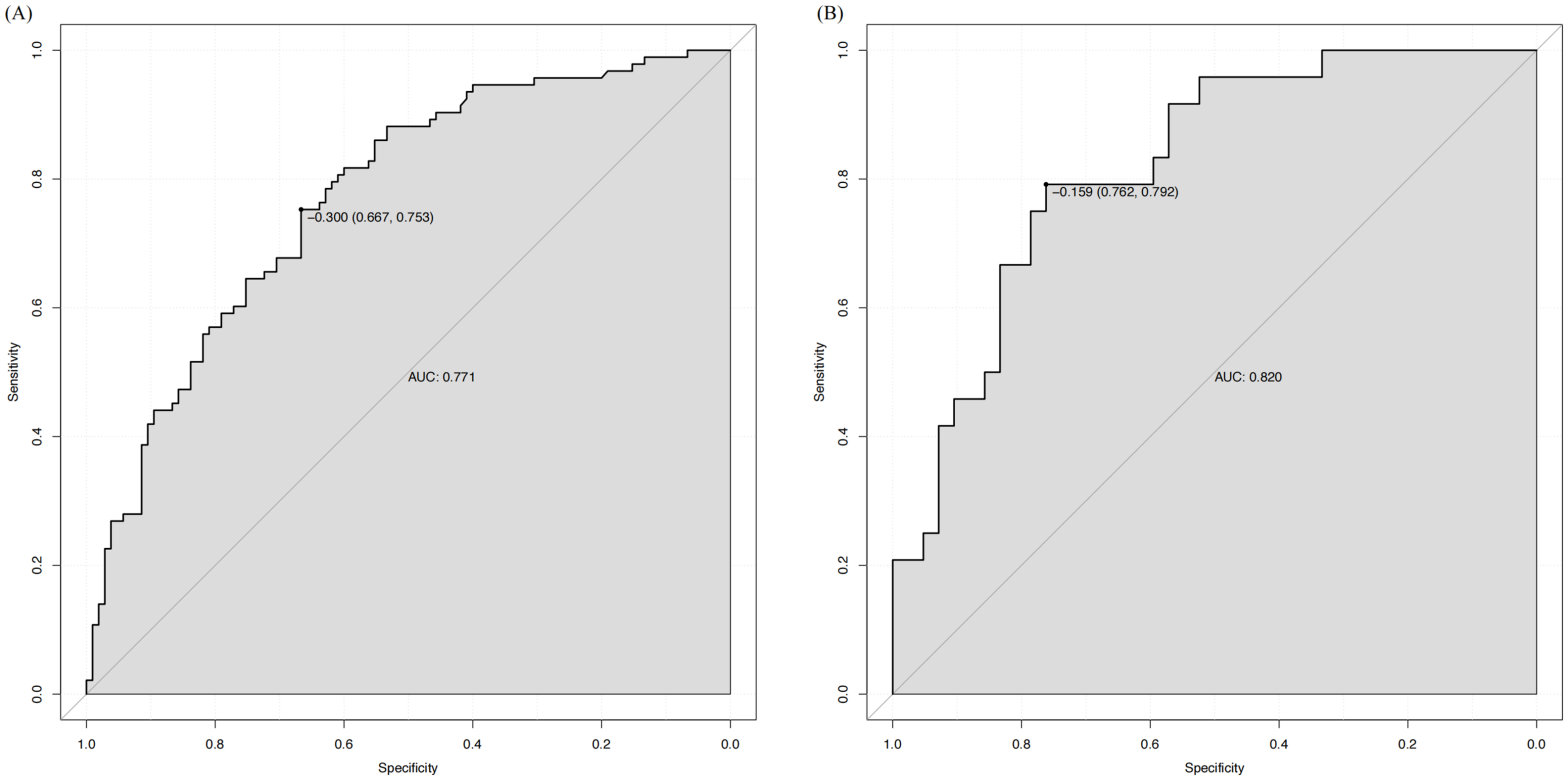
Receiver operating characteristic (ROC) curves of the nomogram. **(A)** The ROC curve for the training cohort with an AUC of 0.771. **(B)** The ROC curve for the validation cohort with an AUC of 0.820.

**Figure 5:**
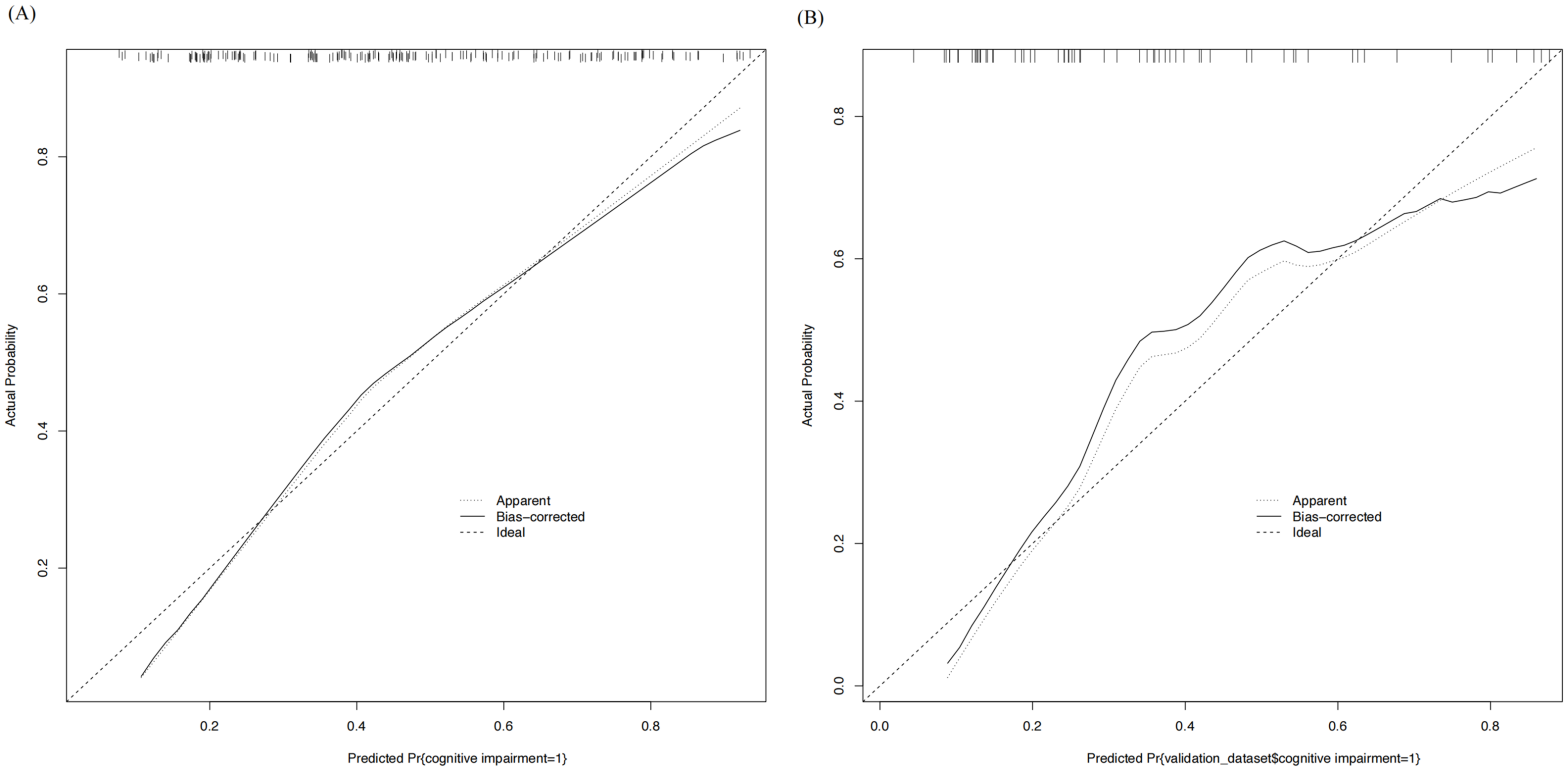
Calibration curves of the nomogram. The diagonal dotted line represents the ideal prediction model. The solid line represents the performance of the nomogram in the training set **(A)** and the validation set **(B)**.

**Figure 6:**
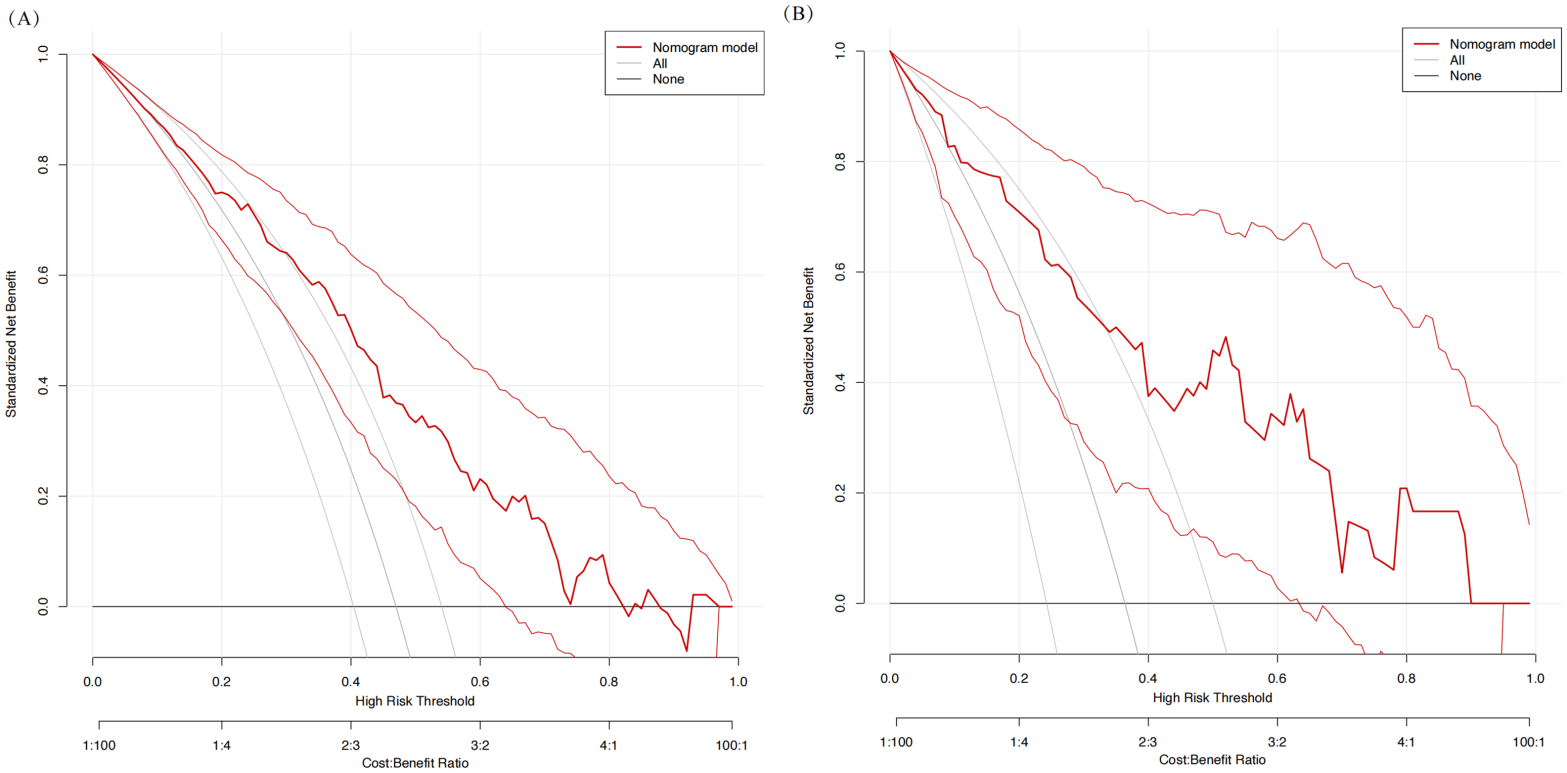
Decision curve analysis (DCA) of the nomogram. The *y*-axis measures the net benefit. The red line represents the nomogram. **(A)** DCA for the training set. **(B)** DCA for the validation set.

Based on the multivariate analysis results, we constructed a prognostic nomogram to assist in personalized risk assessment (Figure 3). By locating a patient’s specific years of education, hematoma volume, and pulmonary infection status on the corresponding variable axes, clinicians can calculate a total score corresponding to a specific probability of cognitive impairment occurrence. The nomogram’s predictive accuracy was validated via ROC analysis (Figure 4). The model achieved an AUC of 0.771 (95% CI: 0.706–0.837) in the training set and a higher AUC of 0.820 (95% CI: 0.726–0.914) in the validation set, indicating stable discriminative ability. In the training set, the AUC of the nomogram (0.771) was significantly higher than that of using pulmonary infection alone (AUC = 0.640, *p* = 0.005), hematoma volume alone (AUC = 0.675, *p* = 0.030), or years of education alone (AUC = 0.654, *p* = 0.012). In the validation set, the AUC of the nomogram (0.820) was also significantly higher than that of pulmonary infection (AUC = 0.607, *p* = 0.012), hematoma volume (AUC = 0.680, *p* = 0.063), and years of education (AUC = 0.790, *p* = 0.361). Calibration curves (Figure 5) demonstrated high consistency between the probabilities predicted by the nomogram and the observed frequencies of cognitive impairment in both datasets. In addition to visual calibration curves, the goodness-of-fit of the nomogram was formally assessed using the Hosmer–Lemeshow test. The test indicated no significant deviation between predicted and observed outcomes in either the training set (*χ*^2^ = 7.24, *p* = 0.512) or the validation set (*χ*^2^ = 5.89, *p* = 0.660), confirming excellent model calibration. The model’s overall discriminative ability, expressed as the concordance index (C-index), was identical to the AUC values (training set: 0.771; validation set: 0.820). Decision curve analysis (DCA) further quantified the clinical utility, demonstrating that the nomogram provided significant net benefit across a wide range of threshold probabilities (approximately 10 to 80%) when compared to the default strategies of intervening for all patients or for none (Figure 6).

### Repeated cross-validation performance

To complement the single split validation and provide a more stable performance estimate, repeated 10-fold cross-validation was conducted on the training cohort (*n* = 198). Across 100 repeats, the nomogram demonstrated consistent discriminative ability, with a mean AUC of 0.763 (standard deviation, SD = 0.032). This result closely aligns with the AUC obtained from the single training set (0.771) and the independent hold-out validation set (0.820), indicating that the model’s performance is robust and not heavily dependent on a particular data partition.

### Bootstrap validation of model stability

Bootstrap internal validation (1,000 resamples) was performed to evaluate the robustness of the parameter estimates from the final multivariate model. The distributions of the odds ratios for pulmonary infection, years of education, and hematoma volume across all bootstrap samples were analyzed. As summarized in Table 4, the original OR estimates from the model (pulmonary infection: 3.028; years of education: 0.868; hematoma volume: 1.030) were all located close to the median (50th percentile) of their respective bootstrap distributions. Furthermore, the 95% confidence intervals derived from the bootstrap percentiles were narrow and contained the original point estimates. These results indicate that the identified predictors are stable and not artifacts of overfitting or sample variability, supporting the reliability of the nomogram’s foundation.

**Table 4:** Bootstrap validation of the multivariate logistic regression model (1,000 resamples).

Predictor	Original OR (95% CI)	Bootstrap median OR (IQR)	Bootstrap 95% CI	Percentile of original OR
Pulmonary infection	3.028 (1.480, 6.166)	3.112 (2.845, 3.402)	1.502, 6.521	48.2%
Years of education	0.868 (0.809, 0.931)	0.871 (0.852, 0.890)	0.803, 0.941	51.1%
Hematoma volume	1.030 (1.014, 1.047)	1.031 (1.021, 1.042)	1.012, 1.051	49.7%

## Discussion

Through in-depth analysis of a sICH patient cohort, this study successfully developed and validated a nomogram prediction model for PSCI. Our findings revealed a PSCI incidence of 44.3% in this cohort, aligning closely with results from Pendlebury and Rothwell (13) and recent meta-analyses of multiple cohorts, reaffirming that cognitive impairment is a major complication following sICH. After rigorous variable screening, we identified pulmonary infection, years of education, and hematoma volume as three independent predictors of cognitive outcome. The model based on these factors demonstrated good discriminative ability and calibration in both training and validation sets. Decision curve analysis further confirmed its potential clinical benefit, outperforming some traditional single-indicator predictions and highlighting the advantage of multi-dimensional factor assessment.

First, pulmonary infection emerged as a strong independent risk factor for PSCI. It is important to address the apparent discrepancy that while the clinical diagnosis of pulmonary infection predicted cognitive decline, the baseline inflammatory biomarkers (WBC, CRP, NLR, PLR) measured at admission did not show significant differences between the two groups. This can be attributed to the temporal characteristics of the data. The laboratory indicators collected within 24 h of onset primarily reflect the acute sterile inflammatory response to the hematoma itself rather than the systemic inflammation caused by subsequent infections. Pulmonary infection typically develops days to weeks into the hospitalization (hospital-acquired pneumonia). Therefore, the initial baseline markers failed to capture the later inflammatory surge associated with the infection. Crucially, unlike non-modifiable factors such as age or genetic background often cited in previous studies, pulmonary infection represents a preventable and treatable complication. This discrepancy points to a clear future research direction: investigating the relationship between infection control protocols in the acute phase and long-term cognitive outcomes. However, the occurrence of the infection itself acts as a “second hit” to the vulnerable brain. This suggests that the delayed systemic inflammatory response and potential hypoxia associated with pneumonia, rather than the initial post-hemorrhagic inflammation alone, may be significantly associated with secondary neurodegeneration and subsequent cognitive impairment (14–18). Regarding metabolic and hemorheological indicators, admission random blood glucose, MCHC, and γ-GT did not differ significantly between groups. Although our institution’s standardized protocol allowed for the uniform collection of these markers (including γ-GT as part of the routine hepatic panel) within 24 h of onset, their exclusion from the final model suggests that transient stress hyperglycemia or baseline metabolic variations may not be as predictive of long-term cognitive outcomes as sustained metabolic dysregulation or the structural damage caused by the hematoma.

However, not all patients experience severe cognitive decline following similar pathological insults (e.g., infection), indicating a crucial buffering role of intrinsic defense mechanisms—specifically, “cognitive reserve.” Our finding that higher education level is a significant protective factor provides strong clinical evidence for the “education-occupation-cognitive reserve” theory proposed by Zhong et al. (19). From a neurobiological perspective, prolonged education and intellectual activity may promote denser neural network construction and synaptic plasticity, enabling the brain to recruit alternative neural circuits to maintain function following hemorrhagic injury. This “brain reserve” effect might even counteract some negative impacts of underlying small vessel disease pathology (20). A prospective cohort study by Gong et al. also found that higher education level was an independent predictor of cognitive recovery in early PSCI patients during follow-up (21). This suggests that for patients with lower education levels—indicating potentially lower cognitive reserve—more intensive cognitive training interventions and closer follow-up monitoring should be implemented during rehabilitation (22).

Although cognitive reserve can provide a certain degree of protection, the primary structural damage of sICH—the volume of the hematoma—remains the physical basis and fundamental cause that determines the cognitive outcome. Our model showed a significant positive correlation between hematoma volume and PSCI risk, consistent with findings from Jiang et al. (23) and Xiong et al. (24). Beyond the direct physical disruption of neuroanatomical structures by the mass effect of the hematoma itself, the association between larger hematoma volume and cognitive decline may be mediated by secondary biochemical injury. Although our study did not quantify specific biomarkers of ferroptosis or iron metabolism, the hematoma volume serves as a macroscopic indicator of the total burden of hemoglobin degradation products. Existing literature suggests that the lysis of red blood cells releases iron, which can trigger oxidative stress and potentially extend injury to perilesional white matter tracts (25, 26). This cascade may eventually disrupt the connectivity of large-scale brain networks essential for cognitive function. Thus, the strong predictive value of hematoma volume in our nomogram likely reflects both the initial mechanical injury and the potential severity of the subsequent secondary neurotoxicity. Furthermore, larger hematomas are often accompanied by more severe cerebral edema and increased intracranial pressure (27, 28), which further compromise global cerebral perfusion and metabolism (21, 26, 29, 30), leading to more extensive disruption of neural network connectivity. Targeting this mechanism, clinical trials of minimally invasive surgery for hematoma evacuation to mitigate secondary injury are underway, offering a potential new avenue for improving cognitive prognosis (31). It is worth noting that bleeding location showed statistical significance in the univariate analysis but was not identified as an independent predictor in the multivariate model. This result suggests that in our specific cohort, the impact of anatomical location may have been outweighed by the robust predictive power of hematoma volume. While large lobar hemorrhages often present with significant volumes, hematoma volume emerged as the more statistically dominant continuous variable in the multivariate selection process. However, this exclusion does not diminish the clinical relevance of neuroanatomical location. Lesions in “strategic” areas, such as the thalamus or specific cortical lobes, can disrupt critical cognitive hubs and networks regardless of size. Furthermore, our study relied on the total MoCA score as the outcome measure. While MoCA is a valuable screening tool, it has limitations in isolating specific cognitive domains. It may be less sensitive to subtle executive dysfunction or processing speed deficits that are characteristic of vascular cognitive impairment in specific brain regions. Therefore, while our nomogram prioritizes hematoma volume for risk calculation, clinicians should not overlook the potential impact of lesion location on specific cognitive trajectories.

Furthermore, it is crucial to contextualize our findings by addressing divergences from established literature. Most notably, advanced age is traditionally regarded as the most robust predictor of cognitive decline in large-scale stroke cohorts, reflecting the depletion of cognitive reserve over time. However, in our multivariate model, age did not emerge as an independent risk factor. This discrepancy highlights a specific advantage of our study design: by focusing on the acute to subacute phase (3 months post-discharge), our data suggest that the magnitude of the “acute hit”—defined by hematoma volume and the “second hit” of pulmonary infection—may override the baseline influence of chronological age in the short term. This implies that our nomogram may be particularly valuable for identifying younger or middle-aged patients who might be overlooked by age-heavy scoring systems but are nonetheless at high risk due to severe initial injury and complications. Similarly, while recent studies have advocated for the prognostic value of admission inflammatory biomarkers (e.g., NLR, PLR), our negative findings regarding these static admission markers—contrasted with the strong predictive value of the clinical event of pulmonary infection—suggest that monitoring the *dynamic* evolution of complications during the hospital course offers superior prognostic utility for cognitive outcomes than relying solely on biomarkers measured at the emergency door.

We also carefully scrutinized variables that reflect the hospital course but are unknown at the time of admission. For instance, length of hospital stay and the need for tracheotomy appeared significant in our initial screening. However, we excluded them from the final prediction model. This decision aligns with the model’s design as an “in-hospital update model” intended for use specifically at discharge. Including outcomes of the stay, such as its duration or procedural requirements, would introduce circular logic. It would also reduce the model’s utility for early planning. Our goal remained to identify baseline and in-hospital complication markers available during hospitalization that predict the post-discharge cognitive outcome.

To enhance the clinical applicability of our findings, we constructed an intuitive nomogram tool, allowing the complex regression equation to be rapidly applied at the bedside. In this study, we developed and validated a simple nomogram model based on admission indicators to predict the development of cognitive impairment in sICH patients. This nomogram performed well in discrimination, calibration, and clinical application, providing valuable information for identifying high-risk patients likely to develop PSCI. Here we provide an example of how to use this nomogram model: suppose a sICH patient has 6 years of education, an admission CT showing a hematoma volume of 35 mL and develops pulmonary infection during hospitalization. According to the nomogram (Figure 3), we mark the score for each parameter on the “Points” axis: [6 years of education (~65 points) + 35 mL hematoma volume (~40 points) + Pulmonary infection “Yes” (100 points) = Total score ~205 points]. Projecting this total score downward, it corresponds to a PSCI risk probability of approximately 85%. This result alerts the clinician that this patient belongs to a very high-risk group, warranting immediate initiation of neuropsychological monitoring, incorporation of an intensive cognitive rehabilitation plan into the discharge strategy, and comprehensive clinical management of complications such as pulmonary infection.

### Limitation

This study has several limitations. First, we observed that the discriminative accuracy (AUC) in the validation set (0.820) was slightly higher than in the training set (0.771). This atypical finding must be interpreted with caution. Observation of the calibration curves reveals that the validation cohort exhibits noticeable overestimation in the intermediate predicted probability range (0.3–0.6). This suggests that while the model is effective at separating low-risk and high-risk patients, the apparent superiority in AUC is likely a stochastic artifact of the small sample size (*n* = 66) and data distribution, rather than a true performance advantage. In smaller datasets, stochastic variation can occasionally result in a data distribution that allows for clearer separation between groups. Therefore, this performance metric should be interpreted with caution and requires confirmation in larger external cohorts. Second, as a single-center retrospective study with a relatively limited sample size, it is susceptible to selection bias, which may restrict the model’s generalizability to populations of different ethnicities or regions. Third, the study primarily focused on admission baseline indicators and did not incorporate genetic markers like APOE genotype or dynamic evolution indicators such as hematoma expansion, potentially omitting some underlying biological predictive information. Furthermore, cognitive assessment relied mainly on the MoCA scale. Although clinically practical, it may be less sensitive to subtle deficits in specific cognitive domains (e.g., executive function, memory) compared to a full neuropsychological test battery. Future improvements should involve conducting multicenter prospective cohort studies and integrating multimodal imaging and liquid biomarkers to further optimize the model’s predictive performance. Finally, it is imperative to distinguish between risk prediction and causal inference. Although our multivariate model adjusted for key confounders, observational data inevitably contain residual confounding. For instance, pulmonary infection is often intrinsically linked to overall stroke severity, aspiration risk, and prolonged immobilization. While we identified infection as a marker of high cognitive risk, this does not confirm a direct causal pathway where treating the infection reverses cognitive decline. The association may partially reflect the “sick brain” being more prone to systemic complications. Thus, our results should be interpreted as identifying a high-risk phenotype rather than validating a specific therapeutic target.

## Conclusion

In summary, this study successfully constructed a nomogram model incorporating clinical complications, sociodemographic characteristics, and imaging indicators. The model identified pulmonary infection, low education level, and large hematoma volume as key predictors of cognitive impairment after sICH, providing a simple and reliable tool for clinical risk stratification and the planning of post-discharge care.

## Data Availability

The original contributions presented in the study are included in the article/Supplementary material, further inquiries can be directed to the corresponding author.

## References

[1] Tsao CW Aday AW Almarzooq ZI Anderson CAM Arora P Avery CL . Heart disease and stroke statistics-2023 update: a report from the American Heart Association. Circulation. (2023) 147:e93–e621. doi: 10.1161/CIR.0000000000001123, 36695182 PMC12135016

[2] GBD 2019 Stroke Collaborators. Global, regional, and national burden of stroke and its risk factors, 1990–2019: a systematic analysis for the Global Burden of Disease Study 2019. Lancet Neurol. (2021) 20:795–820. doi: 10.1016/S1474-4422(21)00252-0, 34487721 PMC8443449

[3] Cordonnier C Demchuk A Ziai W Anderson CS. Intracerebral haemorrhage: current approaches to acute management. Lancet. (2018) 392:1257–68. doi: 10.1016/S0140-6736(18)31878-6, 30319113

[4] GBD 2021 Nervous System Disorders Collaborators. Global, regional, and national burden of disorders affecting the nervous system, 1990–2021: a systematic analysis for the Global Burden of Disease Study 2021. Lancet Neurol. (2024) 23:344–81. doi: 10.1016/S1474-4422(24)00038-3, 38493795 PMC10949203

[5] Potter T Lioutas VA Tano M Pan A Meeks J Woo D . Cognitive impairment after intracerebral hemorrhage: a systematic review of current evidence and knowledge gaps. Front Neurol. (2021) 12:716632. doi: 10.3389/fneur.2021.716632 34512528 PMC8429504

[6] El Husseini N Katzan IL Rost NS Blake ML Byun E Pendlebury ST . Cognitive impairment after ischemic and hemorrhagic stroke: a scientific statement from the American Heart Association/American Stroke Association. Stroke. (2023) 54:e272–91. doi: 10.1161/STR.0000000000000430, 37125534 PMC12723706

[7] Wardlaw JM Smith C Dichgans M. Small vessel disease: mechanisms and clinical implications. Lancet Neurol. (2019) 18:684–96. doi: 10.1016/S1474-4422(19)30079-1 31097385

[8] Westendorp WF Nederkoorn PJ Vermeij JD Dijkgraaf MG van de Beek D. Post-stroke infection: a systematic review and meta-analysis. BMC Neurol. (2011) 11:110. doi: 10.1186/1471-2377-11-110 21933425 PMC3185266

[9] Stern Y Arenaza-Urquijo EM Bartrés-Faz D Belleville S Cantilon M Chetelat G . Whitepaper: defining and investigating cognitive reserve, brain reserve, and brain maintenance. Alzheimers Dement. (2020) 16:1305–11. doi: 10.1016/j.jalz.2018.07.219, 30222945 PMC6417987

[10] Yuan X Xu Q Du F Gao X Guo J Zhang J . Development and validation of a model to predict cognitive impairment in traumatic brain injury patients: a prospective observational study. EClinicalMedicine. (2025) 80:103023. doi: 10.1016/j.eclinm.2024.103023 39850016 PMC11753911

[11] Greenberg SM Ziai WC Cordonnier C Dowlatshahi D Francis B Goldstein JN . 2022 guideline for the management of patients with spontaneous intracerebral hemorrhage: a guideline from the American Heart Association/American Stroke Association. Stroke. (2022) 53:e282–361. doi: 10.1161/STR.0000000000000407 35579034

[12] Pasi M Poggesi A Salvadori E Pantoni L. Post-stroke dementia and cognitive impairment. Front Neurol Neurosci. (2012) 30:65–9. doi: 10.1159/000333412, 22377866

[13] Pendlebury ST Rothwell PM. Prevalence, incidence, and factors associated with pre-stroke and post-stroke dementia: a systematic review and meta-analysis. Lancet Neurol. (2009) 8:1006–18. doi: 10.1016/S1474-4422(09)70236-4, 19782001

[14] Milosevich E Demeyere N Pendlebury ST. Infection, inflammation, and poststroke cognitive impairment. J Am Heart Assoc. (2024) 13:e9130. doi: 10.1161/JAHA.123.033015, 38214255 PMC10926823

[15] Yu X Xiao H Liu Y Dong Z Meng X Wang F. The lung-brain axis in chronic obstructive pulmonary disease-associated neurocognitive dysfunction: mechanistic insights and potential therapeutic options. Int J Biol Sci. (2025) 21:3461–77. doi: 10.7150/ijbs.109261, 40520003 PMC12160515

[16] Iadecola C Anrather J. The immunology of stroke: from mechanisms to translation. Nat Med. (2011) 17:796–808. doi: 10.1038/nm.2399, 21738161 PMC3137275

[17] Moretta P Ambrosino P Lanzillo A Marcuccio L Fuschillo S Papa A . Cognitive impairment in convalescent COVID-19 patients undergoing multidisciplinary rehabilitation: the association with the clinical and functional status. Healthcare. (2022) 10:480. doi: 10.3390/healthcare10030480, 35326958 PMC8950669

[18] Wang Y Chen Y Chen R Xu Y Zheng H Xu J . Development and validation of a nomogram model for prediction of stroke-associated pneumonia associated with intracerebral hemorrhage. BMC Geriatr. (2023) 23:633. doi: 10.1186/s12877-023-04310-5, 37805464 PMC10559607

[19] Zhong T Li S Liu P Wang Y Chen L. The impact of education and occupation on cognitive impairment: a cross-sectional study in China. Front Aging Neurosci. (2024) 16:1435626. doi: 10.3389/fnagi.2024.1435626, 39070104 PMC11273364

[20] Lövdén M Fratiglioni L Glymour MM Lindenberger U Tucker-Drob EM. Education and cognitive functioning across the life span. Psychol Sci Public Interest. (2020) 21:6–41. doi: 10.1177/1529100620920576, 32772803 PMC7425377

[21] Gong L Gu Y Yu Q Wang H Zhu X Dong Q . Prognostic factors for cognitive recovery beyond early poststroke cognitive impairment (PSCI): a prospective cohort study of spontaneous intracerebral hemorrhage. Front Neurol. (2020) 11:278. doi: 10.3389/fneur.2020.00278 32411073 PMC7198781

[22] Ojala-Oksala J Jokinen H Kopsi V Lehtonen K Luukkonen L Paukkunen A . Educational history is an independent predictor of cognitive deficits and long-term survival in postacute patients with mild to moderate ischemic stroke. Stroke. (2012) 43:2931–5. doi: 10.1161/STROKEAHA.112.667618, 22935400

[23] Jiang Q Liu C Zhang H Liu R Zhang J Guo J . Predictors of affective disturbances and cognitive impairment following small spontaneous supratentorial intracerebral hemorrhage. Eur J Neurol. (2025) 32:e16544. doi: 10.1111/ene.16544, 39540700 PMC11625928

[24] Xiong L Reijmer YD Charidimou A Cordonnier C Viswanathan A. Intracerebral hemorrhage and cognitive impairment. Biochim Biophys Acta. (2016) 1862:939–44. doi: 10.1016/j.bbadis.2015.12.011, 26692171

[25] Dixon SJ Lemberg KM Lamprecht MR Skouta R Zaitsev EM Gleason CE . Ferroptosis: an iron-dependent form of nonapoptotic cell death. Cell. (2012) 149:1060–72. doi: 10.1016/j.cell.2012.03.042, 22632970 PMC3367386

[26] Keep RF Hua Y Xi G. Intracerebral haemorrhage: mechanisms of injury and therapeutic targets. Lancet Neurol. (2012) 11:720–31. doi: 10.1016/S1474-4422(12)70104-7, 22698888 PMC3884550

[27] Benedictus MR Hochart A Rossi C Boulouis G Hénon H van der Flier WM . Prognostic factors for cognitive decline after intracerebral hemorrhage. Stroke. (2015) 46:2773–8. doi: 10.1161/STROKEAHA.115.010200, 26272386

[28] Koivunen RJ Harno H Tatlisumak T Putaala J. Depression, anxiety, and cognitive functioning after intracerebral hemorrhage. Acta Neurol Scand. (2015) 132:179–84. doi: 10.1111/ane.12367 25639837

[29] Moulin S Casolla B Kuchcinski G Boulouis G Rossi C Hénon H . Cortical superficial siderosis: a prospective observational cohort study. Neurology. (2018) 91:e132–8. doi: 10.1212/WNL.0000000000005778, 29884737

[30] Moulin S Labreuche J Bombois S Rossi C Boulouis G Hénon H . Dementia risk after spontaneous intracerebral haemorrhage: a prospective cohort study. Lancet Neurol. (2016) 15:820–9. doi: 10.1016/S1474-4422(16)00130-7, 27133238

[31] Hanley DF Thompson RE Rosenblum M Yenokyan G Lane K McBee N . Efficacy and safety of minimally invasive surgery with thrombolysis in intracerebral haemorrhage evacuation (MISTIE III): a randomised, controlled, open-label, blinded endpoint phase 3 trial. Lancet. (2019) 393:1021–32. doi: 10.1016/S0140-6736(19)30195-3, 30739747 PMC6894906

